# ARID2 mutations may relay a distinct subset of cutaneous melanoma patients with different outcomes

**DOI:** 10.1038/s41598-024-54136-3

**Published:** 2024-02-11

**Authors:** Favour A. Akinjiyan, George Nassief, Jordan Phillipps, Tolulope Adeyelu, Andrew Elliott, Farah Abdulla, Alice Y. Zhou, George Souroullas, Kevin B. Kim, Ari Vanderwalde, Soo J. Park, George Ansstas

**Affiliations:** 1 Division of Medical Oncology, Department of Medicine, Washington University in Saint Louis, 4921 Parkview Place, Saint Louis, MO 63110 USA; 2 Caris Life Sciences, Phoenix, AZ USA; 3 California Pacific Medical Center Research Institute, San Francisco, CA USA; 4 Division of Hematology/Oncology, Moores Cancer Center, University of California San Diego, La Jolla, CA USA

**Keywords:** Cancer, Computational biology and bioinformatics

## Abstract

*ARID* genes encode subunits of SWI/SNF chromatin remodeling complexes and are frequently mutated in human cancers. We investigated the correlation between *ARID* mutations, molecular features, and clinical outcomes in melanoma patients. Cutaneous melanoma samples (n = 1577) were analyzed by next-generation sequencing. Samples were stratified by pathogenic/likely pathogenic mutation in *ARID* genes (*ARID1A/2/1B/5B*). PD-L1 expression was assessed using IHC (SP142; positive (+): ≥ 1%). Tumor mutation burden (TMB)-high was defined as ≥ 10 mutations/Mb. Transcriptomic signatures predictive of response to immune checkpoint inhibitors—interferon gamma and T-cell inflamed score were calculated. Real-world overall survival (OS) information was obtained from insurance claims data, with Kaplan–Meier estimates calculated from time of tissue collection until last date of contact. Mann–Whitney U, Chi-square, and Fisher exact tests were applied where appropriate, with *p* values adjusted for multiple comparisons. *ARID2* mutations were more prevalent in cutaneous melanoma compared to *ARID1A* (11.0%: n = 451 vs 2.8%: n = 113), with concurrent *ARID1A*/*ARID2* mutation in 1.1% (n = 46) of samples. *ARID* mutations were associated with a high prevalence of RAS pathway mutations—*NF1* (*ARID1A*, 52.6%; *ARID2*, 48.5%; *ARID1A/2*, 63.6%; and ARID-WT, 13.3%; *p* < 0.0001) and *KRAS* (*ARID1A*, 3.5%; *ARID2*, 3.1%; *ARID1A/2*, 6.5%; and ARID-WT, 1.0%; *p* = 0.018)), although *BRAF* mutations were less common in ARID-mutated cohorts (*ARID1A*, 31.9%; *ARID2*, 35.6%; *ARID1A/2*, 26.1%; and ARID-WT, 50.4%; *p* < 0.0001). TMB-high was more common in ARID-mutated samples (*ARID1A*, 80.9%; *ARID2*, 89.9%; *ARID1A/2*, 100%; and ARID-WT, 49.4%; *p* < 0.0001), while PD-L1 positivity was similar across subgroups (*ARID1A*, 43.8%; *ARID2*, 51.1%; *ARID1A/2*, 52.5%; and ARID-WT, 44.9%; *p* = 0.109). Patients with *ARID1A* mutations had a higher prevalence of dMMR/MSI-H compared to those with ARID-WT (2.7% vs 0.2%, *p* = 0.030). Median IFN-γ and T-cell signatures were higher in *ARID2*-mutated samples compared to ARID-WT (IFN-γ: − 0.15 vs − 0.21, *p* = 0.0066; T-cell: 23.5 vs − 18.5, *p* = 0.041). *ARID2-*mutated patients had improved survival compared to ARID-WT; (HR: 1.22 (95% CI 1.0–1.5), *p* = 0.022). No additional OS benefit was observed with anti-PD-1 therapy for *ARID2* mutation compared to *ARID*-WT. Melanoma patients with *ARID* mutations exhibited higher prevalence of markers associated with ICI response, including TMB-H, and immune-related signatures. Our data also suggests improved survival outcome in patients with *ARID2* mutations, irrespective of anti-PD1 therapy.

## Introduction

Skin cancer is the most common type of cancer in the United States, constituting major health and financial burdens for these patients^1–3^. Melanoma, the most serious form of skin cancer, accounts for 1% of all skin cancers; however, the rates of melanoma have been rising over the last decade^4^. In the USA, melanoma is the 5th most common cancer (in both men and women)^5^, and the American Cancer Society predicts that ~ 97,610 new melanomas will be diagnosed in 2023 (with ~ 7990 deaths)^4^. Despite being the most aggressive cutaneous malignancy, the mortality rate associated with a melanoma diagnosis has declined over the last decade (5% per year in individuals < 50 years old; 3% per year in individuals aged 50 and older), largely attributed to advances in treatment and public education efforts emphasizing early diagnosis and management^4^. Nonetheless, up to 50% of patients do not respond to the newer therapies and thus more research is warranted to identify new targets and treatment options^6^.

Genome sequencing studies of melanoma tumors have identified many mutations in genes that function across a wide spectrum cellular and molecular processes, and which may serve as biomarkers or new targets for therapy. One class of genes frequently mutated in melanoma are epigenetic regulators, such as subunits of the chromatin remodeling complex SWI/SNF (SWItch/Sucrose Non-Fermentable). The SWI/SNF complex is comprised of numerous proteins with the majority of mutations found in the ATPase subunits (SMARCA) and its core components, ARID1A, ARID1B and ARID2^7–9^. The ARID (A-T Rich Interaction Domain) family of proteins is a diverse family of proteins with DNA-binding properties, involved in numerous roles for cellular growth and development and tissue-specific gene expression^10,11^. Within the SWI/SNF complex, ARID proteins contribute to DNA-binding and recruitment of the rest of the complex to genomic regions. The ATPase subunits then mediate nucleosome rearrangement, resulting in altered chromatin accessibility and regulation of gene expression. Despite their DNA-binding capacity, and unlikely typical transcription factors, no specific DNA recognition motif has been identified at ARID binding sites. Mutations in SWI/SNF components are implicated in tumorigenesis among various types of cancer, with specific SWI/SNF subunits distrinctively mutated in different types of cancers, including hepatocellular, colorectal, lung, breast and melanoma cancers^12–19^. Relatedly, current literature supports ARID family proteins in functioning primarily as tumor-suppressors, occasionally engaging in tumor initiation via the PI3K/AKT and Wnt signaling pathway^20–22^. In melanoma, loss of ARID2 in animal models causes global changes in chromatin accessibility and genomic occupancy of melanoma-specific transcription factors, and enhances the ability of melanoma cells to colonize distal organs in animal models^23^.

Immune checkpoint inhibitors (ICIs) are immuno-modulatory therapeutics that have greatly improved cancer treatment and are used extensively for the treatment of melanoma. While many patients respond to immunotherapy, a significant fraction does not. For instance, overall response rate for pembrolizumab in advanced melanoma is 32.9%^24^ The variability in patients’ response highlights the need for better mechanistic understanding of the underlying mutations and improved accuracy of predictive biomarkers for ICI outcomes, such as tumor mutation burden (TMB), PD-L1 expression, and microsatellite instability (MSI)^25–29^. Mutations in epigenetic regulators have previously been associated with different outcomes with immunotherapy treatment. Specifically, in a pan-cancer analysis of 1,660 cancer patients who received ICI therapy, Zhu et. al observed that patients with ARID mutations have higher TMB (associated with increased immunogenicity) and improved overall survival, suggesting that ARID mutations are associated with better response to checkpoint inhibitors and enhanced immune activation^13^. These results were consistent with a previous study showing that aberrations in ARID1A resulted in limited accessibility to IFN-responsive genes and impaired IFN gene expression in ovarian clear cell carcinoma mouse models^30^. ARID proteins are therefore promising novel biomarkers that could predict cancer prognosis and response to ICI treatment^31,32^, and more research is warranted to further characterize this relationship in patients with melanomas. In this study, we assessed a cohort of cutaneous melanoma patients with ARID mutations to identify the ARID2 mutation’s impact on prognosis and response to ICI treatment for cutaneous melanoma patients.

## Methods

### Study cohort

Formalin-fixed paraffin-embedded (FFPE) samples from patients with cutaneous melanoma were submitted to a commercial CLIA-certified laboratory for molecular profiling (Caris Life Sciences, Phoenix, AZ). Samples were analyzed by next-generation sequencing (NGS), whole exome sequencing (WES), whole transcriptome sequencing (WTS), immunohistochemistry (IHC), for molecular and genomic features. The study follows guidelines provided by the Declaration of Helsinki, Belmont Report, and U.S. Common Rule. In accordance with compliance policy 45 CFR 46.101(b), this study was conducted using retrospective, de-identified clinical data, patient consent was not required, and the study was considered IRB exempt by the Washington University Institutional Review Board (IRB ID 202312142). Inclusion criteria are cutaneous melanoma samples tested for ARID1A/2 mutation (Pathogenic or Likely Pathogenic or Wildtype).

### DNA next generation sequencing (NGS)

In preparation of the samples for molecular testing, tumor enrichment was done by harvesting targeted tissues using manual microdissection techniques. Genomic DNA was extracted from FFPE tissue samples and subjected to NGS using the NextSeq or NovaSeq 6000 Platforms (Illumina, Inc. San Diego, CA). A custom SureSelect XT assay (Agilent Technologies, Santa Clara, CA) was utilized to enrich exonic regions 592 whole-gene targets. For tumor sample sequenced on Novaseq 6000 platform, more than 700 clinically relevant genes were assessed. All variants were detected with > 99% confidence based on allele frequency and amplicon coverage, with an average sequencing depth of coverage of > 500 and an analytic sensitivity threshold established of 5% for variant calling. Certified molecular geneticists examined the identified genomic variants and categorized them in alignment with the standards set by the American College of Medical Genetics and Genomics (ACMG). Calculation of mutation frequencies in individual genes included 'pathogenic' and likely pathogenic' variants, while those labeled as 'benign,' likely benign,' and 'variants of unknown significance' were excluded**.**

TMB was measured by counting all non-synonymous missense, nonsense, in-frame insertion/deletion, and frameshift mutations found per tumor that had not been previously described as germline alterations in dbSNP151, Genome Aggregation Database (gnomAD) databases, or benign variants identified by Caris’s geneticists. High TMB (TMB-H) was defined by a cut-off of ≥ 10 mutation/megabase (mut/MB) based on the KEYNOTE-158 pembrolizumab trial, where it was shown that patients with ≥ 10 mut/MB had increased response rates compared to those with < 10 mut/MB^33^. To calculate genomic loss of heterozygosity (LOH), LOH in approximately 250k single nucleotide polymorphisms (SNPs) within segmented autosomal chromosomes was calculated. LOH was based on percentage of all 552 segments with observed LOH (High ≥ 16%, Low < 16%; if fewer than 3000 SNPs were read, the test was reported as indeterminate). We also estimated the Total Neoantigen Load (TNL) as the total number of peptides with predicted binding-level affinity for patient-specific HLA alleles.

### Whole transcriptomic sequencing

Formalin-fixed paraffin-embedded (FFPE) tissue sections mounted on glass slides underwent staining with nuclear fast red (NFR). Regions that contained a minimum of 10% tumor content were delineated for manual microdissection and subsequent mRNA extraction. Whole transcriptome sequencing (WTS) was executed using the Illumina NovaSeq platform (Illumina, Inc., San Diego, CA) along with the Agilent SureSelect Human All Exon V7 bait panel (Agilent Technologies, Santa Clara, CA), and the resulting data reported transcripts per million (TPM).

### Immunohistochemistry (IHC)

IHC was conducted on complete sections of formalin-fixed paraffin-embedded (FFPE) tissues mounted on glass slides. The slides underwent staining employing automated staining methods as directed by the manufacturer. These procedures were meticulously optimized and confirmed to meet the standards outlined by CLIA/CAO and ISO. PD-L1 expression was determined using primary antibody SP142 (Spring Biosciences, Pleasanton, CA, USA), with a positive threshold of ≥ 2 + stain intensity and ≥ 5% percentage of cells stained.

### Immune cell deconvolution and RNA signatures

Immune cell fraction was calculated using the quanTIseq pipeline, which employed deconvolution of bulk transcriptomic data^34^. Interferon (IFN) scores were calculated using a validated 6-gene signature including *IDO1, CXCL10, CXCL9, HLA DRA, STAT1*, and *IFNG*^35^.

### Deficient mismatch repair/microsatellite instability-high (dMMR/MSI-H)

dMMR/MSI-H was determined by a combination of immunohistochemistry (IHC) using antibodies for *MLH1* (M1 antibody), *MSH2* (G2191129 antibody), *MSH6* (44 antibody), and *PMS2* (EPR3947 antibody) from Ventana Medical Systems (Tucson, AZ), and next-generation sequencing (NGS). The outcomes from these three platforms are mostly in agreement, as previously described^36^. In instances where conflicting results emerged, the order of priority for determining the MSI/MMR status of the tumor was IHC, followed by NGS.

### Statistics

Continuous data were assessed using a Mann–Whitney U test, and categorical data were evaluated using Chi-square or Fisher’s exact test, where appropriate. For molecular and immune differences, p values were adjusted for multiple comparisons (false discovery rate corrected *q* value < 0.05 was considered significant while p < 0.05 was considered as trends).

### Outcome data—CODEai

Real-world overall survival (rwOS) information was obtained from insurance claims data and calculated from time of biopsy to last contact. Kaplan–Meier estimates were calculated for the molecularly defined patient cohorts and significance was determined as *p* value of < 0.05.

## Results

### Patient characteristics

A total of 1577 cutaneous melanoma tested for *ARID* gene mutation were examined (Table 1). Of those cases, 61% (n = 967) were *ARID*-WT, while 28.5% (n = 451) had *ARID2* mutation and 7.17% (n = 113) had *ARID1A* mutations. Co-mutation of both *ARID1A* and *ARID2* (*ARID1A/2*) was found in 2.92% (n = 46) of cases. *ARID* mutations were more prevalent in males with cutaneous melanoma compared to females.

**Table 1:** Clinicodemographic data for ARID mutated Cutaneous Melanoma patients used in this study (n = 1577).

	*ARID1A*-Mut	*ARID2*-Mut	*ARID1A/2*-Mut	*ARID*-WT	*p* value
Counts	113	451	46	967	
Median age (range)	70 (24–89)	71 (24–89)	72 (32–89)	66 (14–89)	< 0.00001
Male	65.5% (74)	73.8% (333)	80.4% (37)	61.2% (592)	
Female	34.5% (39)	26.2% (118)	19.6% (9)	38.8% (375)	0.00008

### Mutational landscape in ARID mutated cutaneous melanoma cohorts

**Figure 1:**
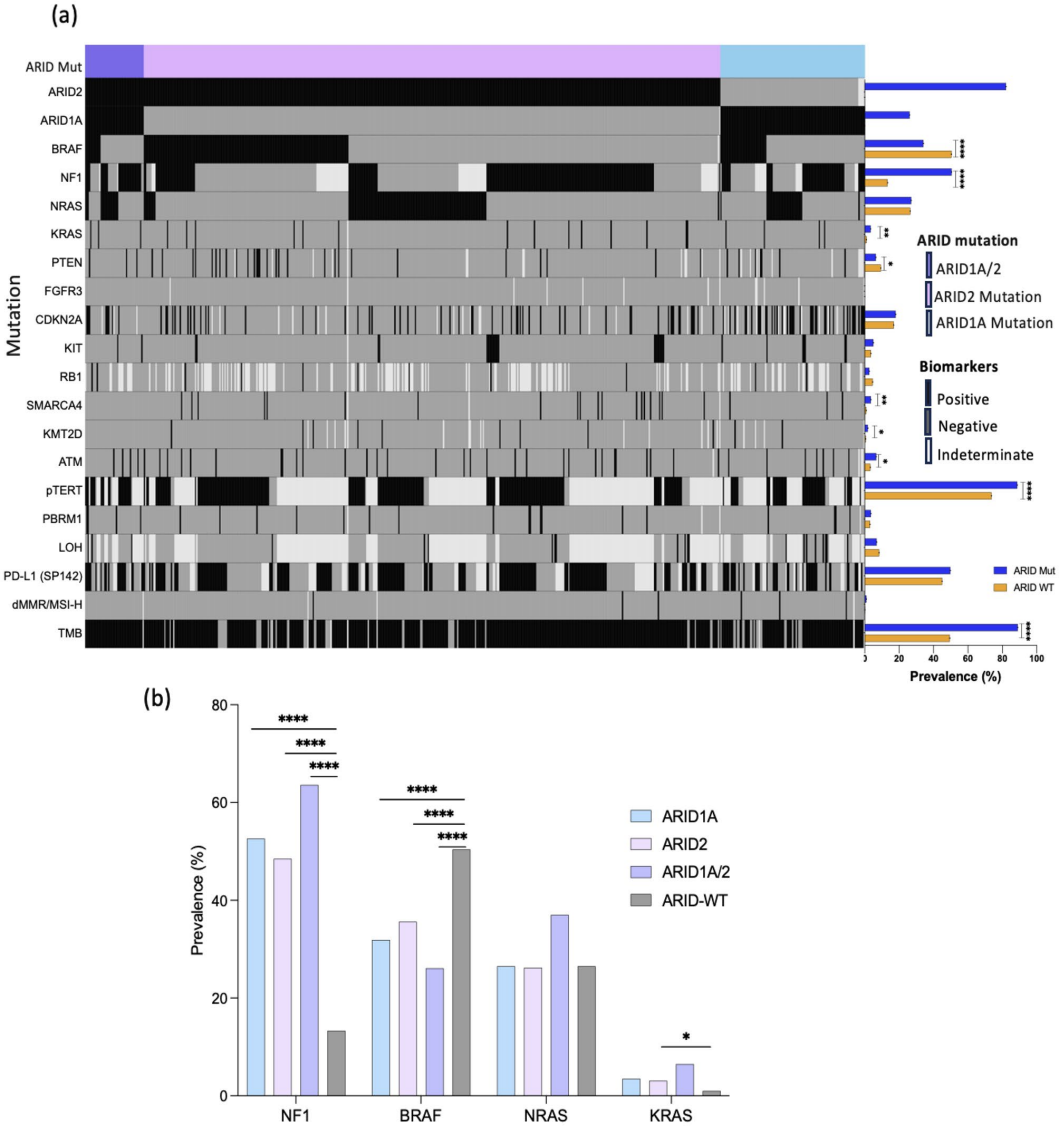
Molecular changes in cutaneous melanoma with *ARID* mutated. (**a**) Oncoprint showing the co-mutations and IO response markers associated *ARID* mutated cutaneous melanoma patients with *ARID* mutations. (**b**) Mutation in MAPK genes in cutaneous melanoma patients harboring *ARID* mutation. Statistical tests performed: Mann–Whitney U test. *q < 0.05, **q < 0.01, ***q < 0.001.

To understand the unique characteristics of ARID mutated cohorts, we analyzed the mutational landscape of genes co-mutated with ARID gene in cutaneous melanoma and compared our observation to ARID-WT patient. The prevalence of *NF1* (50.47% vs 13.31%, *p* < 0.0001), *KRAS* (3.45% vs 1.03%, *q* = 0.0002), *SMARCA4* (3.62% vs 0.93%, *q* = 0.001), *ATM* (6.77% vs 3.31%, *p* = 0.019), *KMT2D* (1.86% vs 0.62%, *p* = 0.022) and *pTERT* (88.67% vs 73.62%, *q* < 0.0001) were significantly higher in the ARID mutated patients compared to ARID-WT. On the other hand, there was a significant decrease in *BRAF* (34.15% vs 50.41%, *q* < 0.0001) and *PTEN* (6.48% vs 9.35%, *q* = 0.047) mutations correlating with ARID mutations. Prevalence of high tumor mutational burden (TMB-H) was significantly associated with *ARID* mutations (88.98% vs 49.42%, *q* < 0.0001) (Fig. 1).

### ARID mutated cutaneous melanoma patients shows alteration in MAPK Associated genes

We analyzed the mutational patterns of mitogen-activated protein kinase (MAPK) associated genes in comparison to the *ARID* mutational status (Fig. 1b). *ARID* mutations were associated with a high prevalence of RAS pathway mutations—*NF1* (*ARID1A*, 52.6%; *ARID2*, 48.5%; *ARID1A/2*, 63.6%; and ARID-WT, 13.3%; *q* < 0.0001) and *KRAS* (*ARID1A*, 3.5%; *ARID2*, 3.1%; *ARID1A/2*, 6.5%; and ARID-WT, 1.0%; *q* = 0.018), although *BRAF* mutations were less common in ARID-mutated cohorts (*ARID1A*, 31.9%; *ARID2*, 35.6%; *ARID1A/2*, 26.1%; and *ARID*-WT, 50.4%; *q* < 0.0001). Majority (> 80%) of the *BRAF* mutation belong to the kinase-activating class 1 and 2 (See supplemental figure 1A).

### Cutaneous melanoma patients with ARID mutations shows comparatively higher immune response markers.

**Figure 2:**
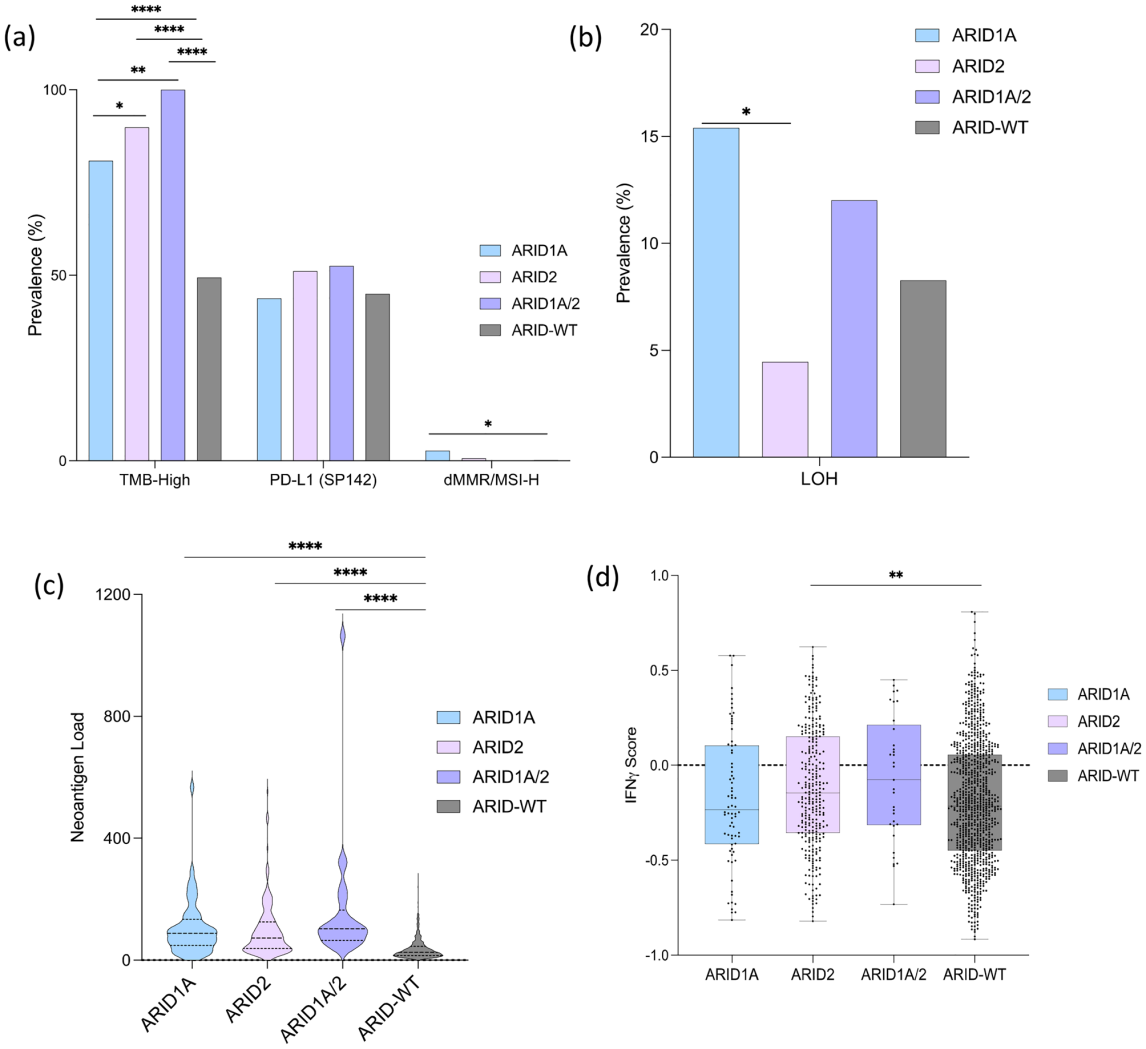
(**a**) Prevalence of Markers predictive of response to immunotherapy (TMB-H, PD-L1 and dMMR/MSI-H) (**b**) Prevalence of loss of heterozygosity in cutaneous melanoma samples. (**c**) Total Neoantigen load for our sample of cutaneous melanoma patients with different *ARID* protein genotypes. (**d**) IFN-γ score in cutaneous melanoma patients with different *ARID* protein genotypes. Statistical test: Mann–Whitney U test. *q < 0.05, **q < 0.01, ***q < 0.001.

Firstly, we examined the association of ARID mutation status with known predictors of immune response markers—TMB-high, PD-L1 and dMMR/MSI-H. ARID mutated cohorts showed a greater prevalence of TMB-high compared to *ARID*-WT (*ARID1A*: 80.9% vs *ARID2*: 89.86% vs *ARID1A*:100% vs *ARID*-WT: 49.42, *q* < 0.00001). The prevalence of dMMR/MSI-H was observed to be significantly higher in patients with ARID1A compared to ARID-WT (2.67% vs 0.21%, *q* = 0.044). Meanwhile, there was no difference in the expression of PD-L1 by IHC in all the cutaneous melanoma cohorts (*ARID1A*: 43.75% vs *ARID2*: 51.08% vs *ARID1A/2*: 52.5% vs *ARID*-WT: 44.96%, *q* = 0.242)—Fig. 2a. We also analyzed the prevalence of loss of heterozygosity in cutaneous melanoma and observed that patients with ARID1A mutation had higher prevalence compared to those with ARID2 mutation (15.38% vs 4.46%, *q* = 0.044)—Fig. 2b.

Furthermore, we measured the total neoantigen load in cutaneous melanoma patients and compared them among patients with ARID mutation. Total Neoantigen Load (TNL) is the total number of peptides with predicted binding-level affinity for patient-specific HLA alleles. We observed that all ARID mutated cohorts have a higher total neoantigen load compared to ARID-WT (Mean TNL: *ARID1A*: 105.4 vs *ARID2*: 96.6 vs *ARID1A/2*: 159.5 vs *ARID*-WT: 37.0, *p* < 0.00001)—Fig. 2c. Lastly, we examined the interferon gamma (IFN-γ) signature—a predictive transcriptomic signature of immune therapy response (IFN-γ score)—^37^. We observed an IFN-γ score higher in the *ARID2* mutation cohort when compared to the WT cohort (Median IFN-γ score: − 0.15 vs − 0.21, *p* = 0.006)—Fig. 2d.

### Tumor microenvironment and immune gene expression in cutaneous melanoma with ARID mutation.

**Figure 3:**
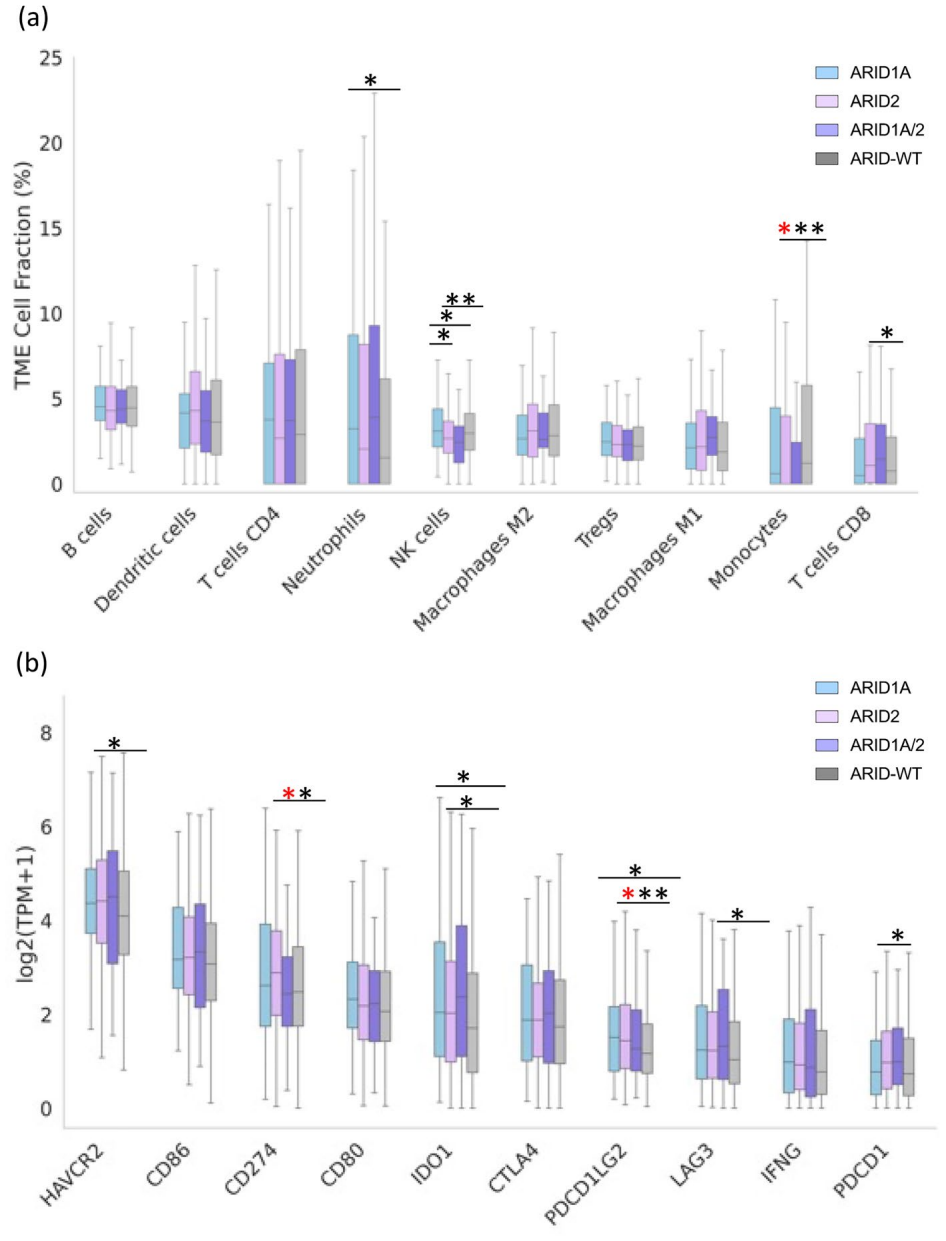
Immune landscape of ARID mutated melanoma. (**a**) Tumor immune microenvironment (**b**) Expression levels of immune checkpoint genes (TPM) in cutaneous melanoma patients with ARID mutation. Statistical significance was noted as: *q < 0.05, *p < 0.05, **p < 0.01.

We investigated the association of *ARID* mutation with the tumor microenvironment and analyzed the immune cell composition (Fig. 3a) in the various cohorts. We observed that the levels of monocytes and natural killer cells were significantly higher in the *ARID*-WT compared to those with *ARID2* mutation (Monocytes Median: 1.2% vs 0.00, *q* = 0.021; NK Cells Median: 2.97% vs 2.67%, *p* = 0.00054). We also observed trends for an increase in T cells—CD8+ (Median: 1.08% vs 0.76%, *p* = 0.021), and myeloid dendritic cells (Median: 4.29% vs 3.63, *p* = 0.022) in *ARID2* mutated cohorts compared to *ARID*-WT. There were also trends for an increase in neutrophils in patients with *ARID1A* mutation compared to *ARID*-WT (Median: 3.23% vs 1.53, *p* = 0.043).

Furthermore, we examine the levels of immune checkpoint genes and compared the impact of *ARID* mutation on the observed changes (Fig. 3b). In specific, patients with *ARID2* mutations showed a significant increase in the expression of immune activation and checkpoint genes, including programed cell death ligand 1—*PD-L1* (*CD274*: 2.89 vs 2.47, *q* = 0.0069), *PD-L2* (*PDCD1LG2*:1.43 vs 1.17, *p* = 0.0050, lymphocytes activation gene—*LAG3* (1.31 vs 1.03, *p* = 0.034), Programmed cell death 1—(*PDCD1*: 0.97 vs 0.73, *p* = 0.0052), hepatitis A virus cellular receptor 2 gene—(*HAVCR2*: 4.40 vs 4.09, *p* = 0.023), and *IDO1* (2.02 vs 1.71, *p* = 0.017), in comparison to the WT cohort. Similarly, the cohort with *ARID1A* mutations exhibited a substantial enrichment in the immune checkpoint *PDCD1LG2* (1.51 vs 1.17, *p* = 0.025) and *IDO1* (2.04 vs 1.71, *p* = 0.024) when compared to the WT cohort.

### Survival analysis for cutaneous melanoma patients with ARID mutations

**Figure 4:**
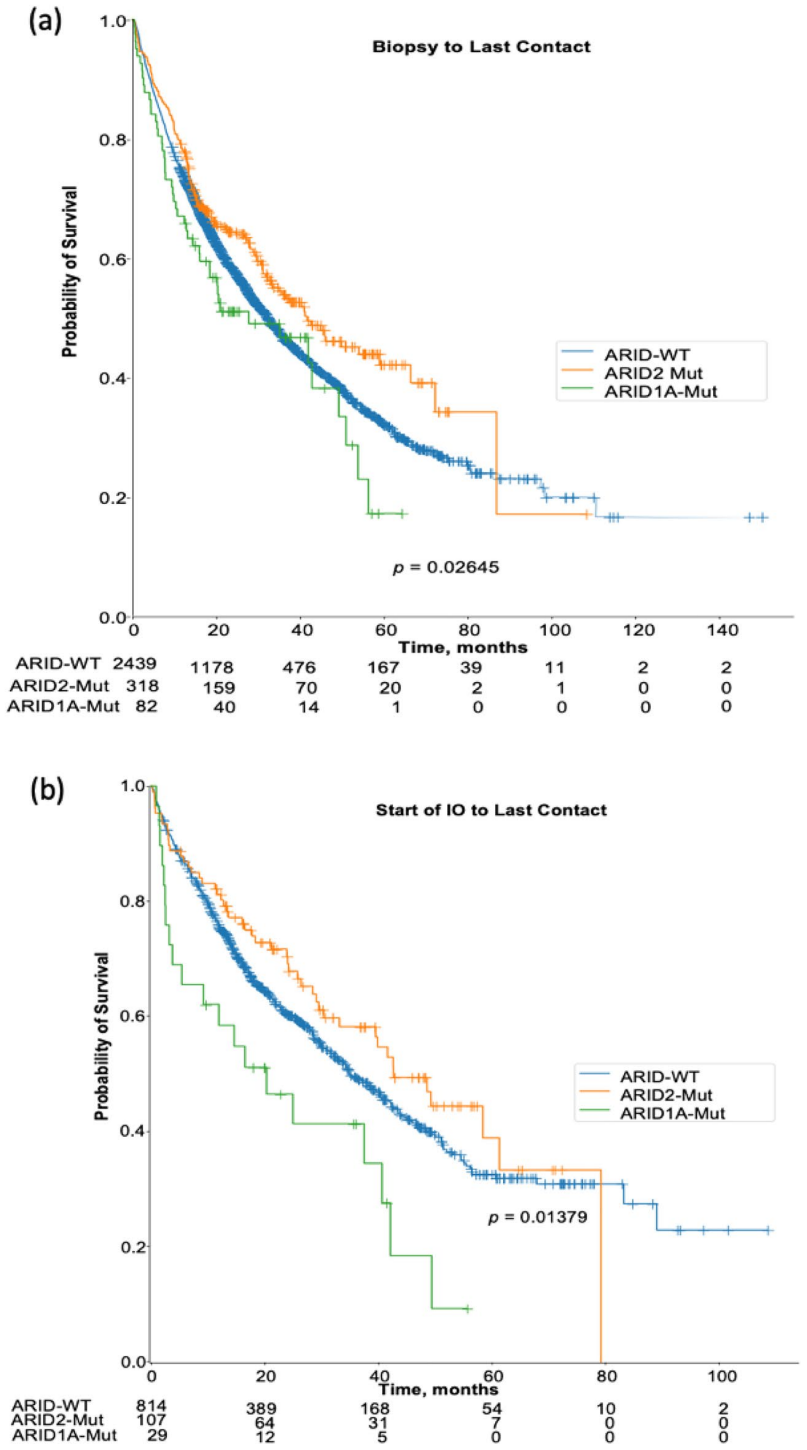
Comparison of the overall survival in *ARID* mutated cutaneous melanoma patients. (**a**) Kaplan Meier curve analysis of the OS among cutaneous melanoma patients with *ARID1A*, *ARID2*—Mutation and ARID-WT from time of biopsy to last contact. (**b**) Kaplan Meier curve analysis of the OS among cutaneous melanoma patients with *ARID1A*, *ARID2*—Mutation and *ARID*-WT from start of immunotherapy to last contact.

We aimed to determine the relationship between *ARID* mutation and real-world patient outcome Fig. 4a,b. We tracked the overall survival (OS) using two metrics—the time of biopsy to the last contact and from the start of immunotherapy to last contact (Table 2). Overall survival was significantly longer in patients with *ARID2* mutation compared to *ARID*-WT (HR 0.82, *p* 0.022), however, this was not significant when we adjusted for treatment with immunotherapy (HR 0.82, *p* = 0.197). Similarly, we observed no difference in the overall survival when we compared patients with *ARID1A* mutation with *ARID*-WT (HR 1.21 *p* = 0.198), however, there was a significant longer OS in *ARID*-WT treated with immunotherapy compared to those with *ARID1A* mutation (HR 1.76, *p* = 0.012). For patients with *ARID2* mutation, treated with PD-1 inhibitors (Pembrolizumab and Nivolumab), there was no statistically significant difference (*p* > 0.05) in the time on treatment when compared to those without *ARID* mutation (Supplemental Figure 2).

**Table 2:** Hazard ratio of the OS in ARID mutated cutaneous melanoma patients.

Outcome	Groups	Median difference	HR	95% CI	*p* value
Biopsy to last contact	*ARID2* versus *ARID*-WT	41.62 versus 32.24	0.82	0.69–0.97	0.022
	*ARID1A* versus *ARID*-WT	27.74 versus 32.24	1.21	0.91–1.62	0.198
Start of IO to last contact	*ARID2* versus *ARID*-WT	42.67 versus 35.04	0.82	0.61–1.11	0.197
	*ARID1A* versus *ARID*-WT	20.39 versus 35.04	1.76	1.12–2.76	0.012

## Discussion

To the best of our knowledge, this is the first study that evaluates ICI response markers with survival outcomes data for cutaneous melanoma patients with various *ARID* mutations. This study highlighted that cutaneous melanoma patients with the *ARID2* mutation showed a distinct profile with a significant association with immunotherapy response markers and survival outcomes.

Cutaneous melanoma patients with *ARID2* mutation showed significant enrichment with immune checkpoint infiltration markers, including high expression of *CD274* (also known as PD-L1), a well-established response marker for immunotherapy^38–40^. Although no statistically significant difference was observed when we examined the direct benefit of anti-PD-1 therapy by measuring the time on treatment with pembrolizumab or nivolumab, the enrichment of several immunotherapy response markers such as high expression of *CD274* and significantly enriched IFN-γ score in patients with ARID2 mutation could relay that they may be particularly responsive to ICI therapy, specifically anti-PD-1 therapy. Ott et. al demonstrated that a higher T-cell inflamed gene expression profile, quantified by IFN- γ score, a high PD-L1 expression level, and a high TMB (stand-alone or in combination) could be utilized as potential predictors for a response for anti-PD-1 therapies^26^. Similarly, Ayers et. al also highlighted that a higher IFN- γ signature is a positive response predictor for anti-PD-1 therapies^35^. Furthermore, the *ARID2* mutation cohort showed trends of increased T-cell CD8+ and myeloid dendritic cell presence within the tumor microenvironment (TME) compared to *ARID*-WT. A higher prevalence of these immune cells within the TME also serves as a positive predictor of immunotherapy response^41^ and tumor prognosis due to their tumor cell dissemination effect^42^. Additionally, Fukumoto et al. had previously demonstrated that T-cells CD8+ and IFN-γ infiltration levels increased significantly in *ARID2* mutation-mice melanoma cells following anti-PD-L1 treatment^43^, further strengthening the argument that *ARID2* mutation patients may be ideal candidates for immunotherapy.

Additionally, we showed that cutaneous melanoma patients with *ARID1A* mutation were enriched in high expression levels of immune checkpoint infiltration genes, such as *PDCD1LG2* and *IDO1*. Despite a comparatively high prevalence of TMB and total neoantigen load in all *ARID* mutated cutaneous melanoma patients, those with *ARID1A* fail to demonstrate a significant increase in IFN-γ score or enrichment in *CD274*, potential markers for ICI therapy response, when compared to WT patients. This is similar to the results from Thielmann et al. where patients with *ARID1A* mutations, despite showing enrichment in PD-L1, high TMB, and impaired mismatch repair (MMR), did not exhibit significantly better outcomes following immunotherapy when compared to patients without the mutation^44^. These findings are consistent with our work and highlight the unique profile of the *ARID2* mutation cutaneous melanoma patients.

Our real-world Overall Survival (rwOS) revealed a significantly higher prognostic advantage in the *ARID2* cohort when compared to *ARID*-WT, however, this was not significant when we compared the time when immunotherapy treatment was started to the last contact. Although our outcomes data do not address the absolute clinical benefit of anti-PD1 therapy in *ARID2*-mutant patients, the rwOS in combination with the increased prevalence of ICI therapy response markers suggest that this cohort may be a distinct group in terms of immunotherapy treatment response. Our data also highlights the distinct profile of patients with *ARID2* mutation compared to those with *ARID1A* as we observed a lower overall survival in the *ARID1A* group when compared to WT from the time of biopsy to last contact, which could indicate this mutation as a biomarker for poor prognosis.

In conclusion, our work presents cutaneous melanoma patients with *ARID2* mutation as potential candidates for ICI therapy and suggests the possibility of improved overall tumor prognosis. However, more clinical outcomes data is needed to demonstrate whether this distinct patient group exhibits better outcomes specifically in response to ICI therapy. It is also unclear how other treatment methods previously administered could synergically affect the patient’s outcomes following ICI therapy. Nevertheless, the correlation between immunotherapy response markers and the significant difference in outcomes among the different patient cohorts make a strong case for considering *ARID2* mutation cutaneous melanoma patients as ideal candidates for immunotherapy.

### Supplementary Information

Supplementary Figures.

## Data Availability

Data is from clinical care of patients and via collaboration with CARIS Life Sciences. The datasets used and/or analyzed during the current study available from the corresponding author on reasonable request.
