# Supplementary material for: ARID2 mutations may relay a distinct subset of cutaneous melanoma patients with different outcomes
Source: Sci Rep. 2024 Feb 11;14:3444. doi: 10.1038/s41598-024-54136-3 (PMC10858967; doi:10.1038/s41598-024-54136-3)
Supplement: Supplementary file 1 — Supplementary Figures. [file 41598_2024_54136_MOESM1_ESM.docx]

Supplemental Figures

**Supplemental Figure 1**: Prevalence of BRAF mutation classes in melanoma patients with ARID mutation. High prevalence of class1/2 BRAF (kinase-activating), however, those with co-mutation of ARID1A/2 had comparatively higher prevalence of class 3 BRAF (BRAF-kinase dead).


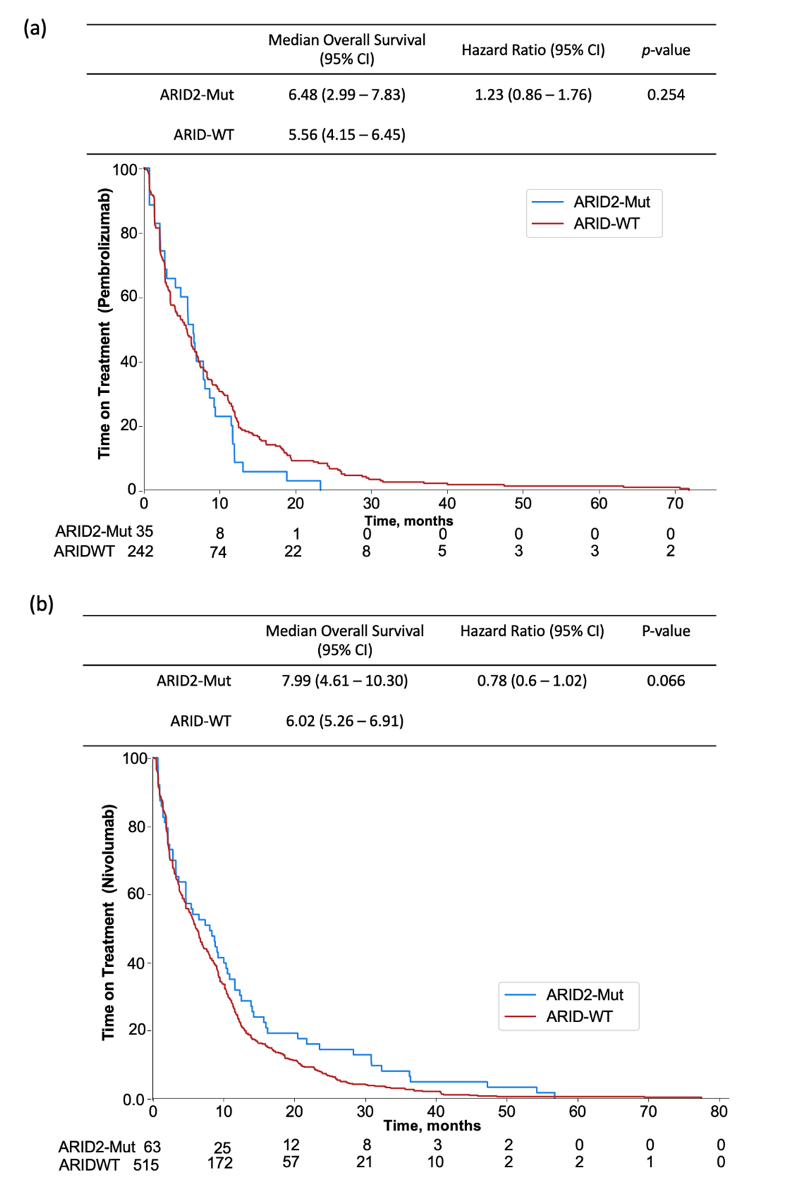


**Supplemental Figure 2**: Time on treatment on PD-1 inhibitors (Pembrolizumab and Nivolumab). No statistically significant improvement on time on treatment with (a) pembrolizumab (b) nivolumab between ARID2 mutated cutaneous melanoma patients and ARID-WT
